# Supplementary material for: Systemic Therapy and Its Surgical Implications in Patients with Resectable Liver Colorectal Cancer Metastases. A Report from the Western Canadian Gastrointestinal Cancer Consensus Conference
Source: Curr Oncol. 2022 Mar 8;29(3):1796–807. doi: 10.3390/curroncol29030147 (PMC8947455; doi:10.3390/curroncol29030147)
Supplement: Supplementary file 1 [file curroncol-29-00147-s001.zip › curroncol-1595031-supplementary.pdf]

Current Oncology

MDPI

Supplementary Materials

Systemic Therapy and Its Surgical Implications in Patients with Resectable Liver Colorectal Cancer Metastases. A Report from the Western Canadian Gastrointestinal Cancer Consensus Conference

Shahid Ahmed, Nicholas Bosma, Michael Moser, Shahida Ahmed, Bryan Brunet, Janine Davies, Corinne Doll, Dorie-Anna Dueck, Christina A. Kim, Shuying Ji, Duc Le, Richard Lee-Ying, Howard Lim, John Paul McGhie, Karen Mulder, Jason Park, Deepti Ravi, Daniel J. Renouf, Devin Schellenberg, Ralph P.W. Wong and Adnan Zaidi

Table S1. List of participants from the Western Canadian Gastrointestinal Cancers Group.

Name	Organization	Discipline	Province
Adnan Zaidi	Saskatchewan Cancer Agency	Medical Oncology	SK
Alec Watson	Arnie Charbonneau Cancer Institute	Medical Oncology	AB
Anahita Dehmoobed	CancerCare Manitoba	Pharmacy	MB
Benjamin Goldenberg	CancerCare Manitoba	Medical Oncology	MB
Bryan Brunet	Saskatchewan Cancer Agency	Radiation Oncology	SK
Chen Zhou	University of British Columbia	Pathology	BC
Christina Kim	CancerCare Manitoba	Medical Oncology	MB
Delia Sauciuc	BC Cancer Agency	Medical Oncology	BC
Duc Le	Saskatchewan Cancer Agency	Radiation Oncology	SK
Edward Hardy	BC Cancer Agency	Medical Oncology	BC
Elvira Planincic	CancerCare Manitoba	Nursing	MB
Georgia Geller	BC Cancer Agency	Medical Oncology	BC
Haji Chalchal	Saskatchewan Cancer Agency	Medical Oncology	SK
Hatim Karachiwala	Cross Cancer Institute	Medical Oncology	AB
Howard Pai	BC Cancer Agency	Radiation Oncology	BC
Jacob Easaw	Cross Cancer Institute	Medical Oncology	AB
James Paul	CancerCare Manitoba	Medical Oncology	MB
Janine Davies	BC Cancer Agency	Medical Oncology	BC
Jennifer Rauw	BC Cancer Agency	Medical Oncology	BC
Jennifer Spratlin	Cross Cancer Institute	Medical Oncology	AB
Jiti Gill	BC Cancer Agency	Medical Oncology	BC
John Shaw	University of Saskatchewan	Hepatobiliary Surgery	SK
Julie Price Hiller	Cross Cancer Institute	Medical Oncology	AB
Kamal Haider	Saskatchewan Cancer Agency	Medical Oncology	SK
Karen King	Cross Cancer Institute	Medical Oncology	AB
Karen Mulder	Cross Cancer Institute	Medical Oncologgy	AB
Kathi Klapp	CancerCare Manitoba	Nursing	MB
Keith Tankel	Cross Cancer Institute	Radiation Oncology	AB
Kelly Cheung	CancerCare Manitoba	Pharmacy	MB
Kimberley Johnson	Alberta Health Services	Nutrition	AB
Lyly Le	BC Cancer Agency	Medical Oncology	BC
Nick Bosma	BC Cancer Agency	Medical Oncology	BC
Mohamed Emara	Saskatchewan Cancer Agency	Oncology	SK
Muhammad Zulfiqar	BC Cancer Agency	Medical Oncology	BC
Mussawar Iqbal	Saskatchewan Cancer Agency	Medical Oncology	SK

Mike Moser	University of Saskatchewan	Hepatobiliary Surgery	SK
Patricia Tang	Arnie Charbonneau Cancer Institute	Medical Oncology	AB
Raida Khwaja	University of Alberta	Medical Oncology	AB
Richard Lee Ying	Arnie Charbonneau Cancer Institute	Medical Oncology	AB
Safiya Karim	Arnie Charbonneau Cancer Institute	Medical Oncology	AB
Sangjune Lee	Arnie Charbonneau Cancer Institute	Radiation Oncology	AB
Shahid Ahmed	Saskatchewan Cancer Agency	Medical Oncology	SK
Sharlene Gill	BC Cancer Agency	Medical Oncology	BC
Shaun Loewen	Arnie Charbonneau Cancer Institute	Radiation Oncology	AB
Shazia Mahmood	Saskatchewan Cancer Agency	Radiation Oncology	SK
Sheryl Koski	Cross Cancer Institute	Medical Oncology	AB
Shuying Ji	Shared Health Manitoba	Pathology	MB
Sunil Parimi	BC Cancer Agency	Medical Oncology	BC
Theresa Chan	BC Cancer Agency	Medical Oncology	BC
Tirath Nijjar	Cross Cancer Institute	Radiation Oncology	AB
Vallerie Gordon	CancerCare Manitoba	Medical Oncology	MB
Wei Xiong	University of British Columbia	Pathology	BC
Will Jiang	BC Cancer Agency	Radiation Oncology	BC

AB=Alberta, BC=British Columbia, MB=Manitoba, SK=Saskatchewan.

Copyright: © 2022 by the authors. Licensee MDPI, Basel, Switzerland. This article is an open access article distributed under the terms and conditions of the Creative Commons Attribution (CC BY) license (https://creativecommons.org/licenses/by/4.0/).
